# Wealth-based equity in maternal, neonatal, and child health services utilization: a cross-sectional study from Ethiopia

**DOI:** 10.1186/s12939-019-1111-2

**Published:** 2019-12-23

**Authors:** Alem Desta Wuneh, Araya Abrha Medhanyie, Afework Mulugeta Bezabih, Lars Åke Persson, Joanna Schellenberg, Yemisrach Behailu Okwaraji

**Affiliations:** 1 School of Public Health, College of Health Sciences, Mekelle University, Mekelle, Ethiopia; 2 London School of Hygiene &Tropical Medicine, London, UK; 3 Ethiopian Public Health Institute, Addis Ababa, Ethiopia

**Keywords:** Wealth-based equity, Maternal health services utilization, Child health services utilization, Wealth quintile, Concentration index, Slope index of inequity, Ethiopia

## Abstract

**Background:**

Despite the pro-poor health policies in Ethiopia, the utilization of maternal, neonatal, and child health services remains a challenge for the country. Health equity became central in the post-2015 Sustainable Development Goals globally and is a priority for Ethiopia. The aim of this study was to assess equity in utilization of a range of maternal and child health services by applying absolute and relative equity indices.

**Methods:**

Data on maternal and child health utilization emanated from a baseline survey conducted for a large project ‘Optimizing the Health Extension Program from December 2016 to February 2017 in four regions of Ethiopia. The utilization of four or more antenatal care visits; skilled birth attendance; postnatal care within 2 days after childbirth; immunization with BCG, polio 3, pentavalent 3, measles and full immunization of children aged 12–23 months; and vitamin A supplementation for 6–23 months old children were stratified by wealth quintiles. The socioeconomic status of the household was assessed by household assets and measured by constructing a wealth index using principal component analysis. Equity was assessed by applying two absolute inequity indices (Wealth index [quintile 5- quintile 1] and slope index of inequality) and two relative inequity indices (Wealth index [quintile5: quintile1] and concentration index).

**Results:**

The maternal health services utilization was low and inequitably distributed favoring the better-off women. About 44, 71, and 18% of women from the better-off households had four or more antenatal visits, utilized skilled birth attendance and postnatal care within two days compared to 20, 29, and 8% of women from the poorest households, respectively. Skilled birth attendance was the most inequitably distributed maternal health service. All basic immunizations: BCG, polio 3, pentavalent 3, measles, and full immunization in children aged 12–23 months and vitamin A supplementation were equitably distributed.

**Conclusion:**

Utilization of maternal health services was low, inequitable, and skewed against women from the poorest households. In contrast, preventive child health services were equitably distributed. Efforts to increase utilization and reinforcement of pro-poor and pro-rural strategies for maternal, newborn and immunization services in Ethiopia should be strengthened.

## Background

Health and health care services are inequitably distributed between and within countries [1]. Wealth-based inequities in the utilization of maternal, neonatal and child health (MNCH) services exist in low-income countries [2]. An analysis in Sub-Saharan Africa (SSA) showed that countries with an improved equitable utilization in maternal and child health services had demonstrated a continued reduction in maternal and child mortality, though inequities in utilization still persisted [3]. These patterns in the utilization of maternal, neonatal, and child health services are also found in the Ethiopian context [4].

In this study, equity is defined based on the concept of equal utilization of services for equal needs [1, 5]. Evidences show that women and children in disadvantaged population subgroups have lower coverage of preventive services and utilization of health care and worse health outcomes than the more advantaged [6, 7]. As a result, the poor suffer higher rates of morbidity and mortality than the better-off and often use health services less despite higher needs [8]. These inequities are serious public health concerns with social and economic implications [6–8]. Thus, health equity became central in the post-2015 Sustainable Development Goals (SDGs) globally [9] and it is also a priority for Ethiopia [10].

Ethiopia has implemented pro-poor health policy through a number of health programs that have demonstrated considerable improvements in maternal and child health services, such as health extension program, health development army, scale-up of community-based health insurance schemes [4, 11]. Existing evidence has, however, shown high levels of inequity in the MNCH services utilization favoring the better-off population subgroups [12, 13]. Nevertheless, few studies have found an equitable distribution in the coverage of immunization across all socioeconomic groups [14].

However, previous studies have demonstrated mixed findings regarding equity in the utilization of MNCH services in Ethiopia [4, 12, 15], also varying by equity measurement used and indices of equity analysis applied. In most of these studies, equity was assessed by applying a single index [4, 16]. This study has used a combination of indices to provide a comprehensive picture of equity in the utilization of services, which is highly recommended [17]. There has been a dynamic and rapid development in socio-economic conditions in Ethiopia in the last couple of decades that may have influenced the utilization of maternal, neonatal, and child health services [18, 19]. Such transitions may also influence the distribution of health services utilization. Thus, the aim of this study was to assess equity in the utilization of a range of maternal and child health services by applying absolute and relative equity indices.

## Methods

### Study setting and design

The study was conducted in ten zones of four regions in Ethiopia, namely Amhara, Oromia, Southern Nations, Nationalities and Peoples (SNNP), and Tigray. These regions cover more than 85% of the total population of the country. Agriculture is the predominant source of economy in these regions. Ethiopia has one of the lowest income per capita but is one of the fastest-growing economies in Africa with an increment in Gross Domestic Product of 7% since 2014 [19]. The health care system is three-tiered: primary, secondary, and tertiary-level of care.

### Data source

The present study was part of an evaluation of the large project entitled “Optimizing the Health Extension Program” (OHEP) that aimed at improving services utilization of the integrated community case management of childhood illnesses (ICCM) and the community-based newborn care (CBNC). The beneficiaries of the intervention are the caretakers and their children under five years, who are the study subjects of this survey. The evaluation of the intervention has been registered in the trial registration Current Controlled Trials ISRCTN12040912. The data were collected from December 2016 to February 2017. A two-stage stratified cluster sampling technique was used to select the study subjects. First, 200 Enumeration Areas (EAs) were selected in the study areas. Each enumeration area formed one cluster that was the primary sampling unit. Second, all households, as secondary sampling units, within a cluster were listed as a sampling frame. Of the listed households, 30 were selected using systematic random sampling. Overall, a total of 6000 households were sampled but data were collected from 5714 households. Data from six EAs in the SNNP region were not collected for security reasons.

### Measurements

Nine indicators were used to assess equity in the utilization of MNCH services. The first three were maternal indicators: four or more antenatal care visits (ANC4+), skilled birth attendance (SBA), and postnatal care visits within two days (PNC). These maternal indicators were based on interviews with women who had a live birth in the past 12 months before the survey. Skilled birth attendance was defined as a dichotomous variable where mothers were coded as having delivered by a skilled birth attendant if they received delivery care by skilled health attendant [20]. Child immunization indicators included BCG, three polio immunizations, three pentavalent immunizations, measles and full immunization of children 12–23 months of age. Information on vitamin A supplementation included children aged 6–23 months [7, 12]. Utilization for all immunization types was based on the combined information recorded on the child’s vaccination card with responses from the caretaker in case of missing immunization card information. Full immunization coverage was defined as the percentage of children aged 12–23 months, who had received one dose of BCG vaccine, three doses of the polio vaccine, three doses of pentavalent vaccine, and one dose of measles vaccine [21]. Utilization of vitamin A supplementation was defined as children aged 6–23 months who had received vitamin A supplementation in the six months preceding the survey. These variables were coded as 1 when the respondent or child had received the service and 0 when the respondent had not received the service.

### Construction of socioeconomic status

The socioeconomic status of each household was constructed using principal component analysis (PCA) of household assets followed by stratification of the households into wealth quintiles [22]. The analysis was done by aggregating the ownership of durable assets; access to utilities and infrastructure; and housing characteristics; ownership of a house and ownership of livestock variables into a single proxy variable of household wealth. All asset variables were coded into binary variables. Asset variables with zero standard deviations were excluded from the PCA as they did not contribute to the analysis. The first component of the PCA was used to construct the wealth quintiles [22]. Based on the PCA weights for each asset variable, an aggregated score was calculated for each of the surveyed households, which was grouped into quintiles with quintile 1 (Q1) representing the poorest 20% of households in the sample and quintile 5 (Q5) representing 20% of the better-off. The study subjects were thereafter grouped into quintiles based on their household wealth scores.

### Equity analyses

Two absolute and two relative equity measures were used to assess equity in the utilization of maternal, neonatal and child health services based on household wealth quintiles. For a given health indicator, the absolute inequity reflects the magnitude of absolute difference in health services utilization between the two subgroups (Q5-Q1) and the slope index of inequity (SII) across the entire distribution of socioeconomic status. The relative inequity measures the ratio (Q5: Q1) and the concentration index (CIX) in health services utilization. The CIX and SII, including standard errors [12] and *p*-values, were assessed using commands downloaded from the International Center for Equity in Health [17, 23]. The utilization of the services by the wealth quintiles and the distance between the wealth quintiles were graphically depicted using equiplot [12, 24]. STATA 14.1 (StataCorp, Texas, USA) was used to analyze the data. During the analysis, all the commands were preceded with svy to account for the clustering.

## Results

### Participation of study subjects

Data were collected from 5714 rural households. A total of 6321 women aged 13 to 49 years old participated in the study with a response rate of 95%. Most of the women (57%) were Christian Orthodox followers and about 46% of them had not attended formal education. Thirty-seven percent of the women were in the age group of fewer than 20 years and 38% were in the age group of 20–34 years. Of the 6321 women, 714 had a live birth in the last 12 months prior to the survey. There were 3110 children under the age of five years of whom 567 (18%) were in the age interval of 12–23 months, while 51% were girls.

### Ownership of assets

Table 1 describes the ownership of the durable assets, utilities and housing characteristics by wealth quintiles. As expected, the ownership of durable assets increased by wealth quintiles, i.e., the better-off households were more likely to own durable assets than the poorest. In addition, the houses from the better-off quintiles were characterized by having an improved water source and an improved latrine. The poorest quintiles were less likely to have an iron roof, but more likely to own the houses where they lived, and more likely to own agricultural land, compared to the better-off.

**Table 1:** Percent of households (*n* = 5714) with specific durable assets and utilities by wealth quintile

Assets and utilities	Q1	Q2	Q3	Q4	Q5	Overall
Ownership of durable assets						
Wristwatch	16	18	21	26	27	22
Gold	0	2	3	8	25	8
Mobile phone	20	42	58	77	89	57
Radio	15	20	30	44	47	31
Bed	13	41	66	76	84	56
Housing characteristics and utilities						
Electricity	0	0	2	13	73	18
Kerosene lamp	17	14	14	12	9	13
Solar energy	52	47	41	38	27	41
Electricity for cooking	0	0	0	0	17	3
Iron roof	26	54	75	88	97	68
Improved toilet facility	43	72	81	89	94	76
Improved source of water	30	54	73	86	95	68
Ownership of house, land, and livestock						
Own house	100	100	99	94	65	92
Own agriculture land	96	91	87	77	40	78
Own livestock	81	72	69	54	25	60

### Maternal health services utilization

Thirty percent of the women had four or more antenatal care visits, 48% had a skilled attendant at their most recent childbirth and just 12% had a postnatal care visit within two days after birth (Table 2 and Fig. 2).

**Table 2:** Wealth-based inequities in utilization of selected maternal and child health services

Type of child health services	No of subjects	Overall utilization	Q1 utilization	Q5 utilization	Difference (Q5-Q1; % points)	Slope index of inequity /SII (% points)			Ratio (Q5: Q1)	Concentration index/CIX (X100)		
	n	%	%	%	%	%	SE	*P*	value	%	SE	P
Maternal health services												
Antenatal care (4 or more visits)	714	30	20	44	24	22.0	5.8	< 0.001	2.2	13.2	3.3	< 0.001
Skilled birth attendance	714	48	29	71	42	46.0	5.4	< 0.001	2.5	17.5	2.1	< 0.001
Postnatal care (within two days)	714	12	8	18	10	9.0	4.2	0 .042	2.2	14.1	6.0	0.020
Child health services												
BCG immunization	567	66	69	65	−4	−9.5	6.8	0.163	0.94	−2.4	1.7	0.170
Polio 3 immunization	567	64	66	63	−3	−6.1	6.9	0.380	0.95	−1.8	1.8	0.338
Pentavalent 3 immunization	567	54	56	57	−1	− 1.7	7.2	0.810	1.02	−0.3	2.2	0.911
Measles immunization	567	61	60	57	−3	−7.0	7.2	0.326	0.95	−2.1	1.9	0.286
Full immunization	567	41	42	44	−2	−5.0	7.2	0.488	1.05	−1.8	3.0	0.548
Vitamin A supplementation	924	57	60	57	−3	− 5.1	5.6	0.361	0.95	−0.7	1.6	0.662

Immunization coverage was computed for children aged 12–23 months and vitamin A supplementation for 6–23 months old. CIX- concentration index, SII-slope index of inequity. SE-standard error, Q1-wealth quintile 1 (20% poorest), Q5-wealth quintile 5 (20% better-off), 95%CI- 95% confidence interval. “Difference” (Q5-Q1) is a measure of an absolute difference in utilization of the services between the better-off (Q5) and poorest (Q1); “Ratio” is a measure of relative inequity of Q5 divided by Q1. The SII is an estimate of absolute inequity that takes the whole population into account and interpreted as the percentage point difference. The CIX is an estimate of relative inequity which takes the whole population into account. For all equity indices, the results are presented in percentages.

There were differences in the utilization of maternal health services between the better-off and poorest population subgroups. The utilization of ANC4+ visits, SBA, and PNC were 20, 29, and 8%, in the poorest wealth quintile and 44, 71, and 18%, respectively, in the better-off wealth quintile. This resulted in a wide absolute inequity gap (Q5-Q1) between the better-off and poorest wealth quintiles for SBA (42 percentage points) and moderate for ANC4+ visits (24 percentage points). Postnatal care had a more limited inequity gap (10 percentage points) (Table 2 and Fig. 2). These findings imply that there were wide differences in the utilization of these three services across the wealth quintiles favoring the better-off. Also, the values of the SII for ANC4+ visits (22 percentage points), SBA (46 percentage points) and PNC (9 percentage points) (Table 2) indicate that the utilization of maternal services was concentrated to the better-off wealth quintiles.

**Fig. 1:**
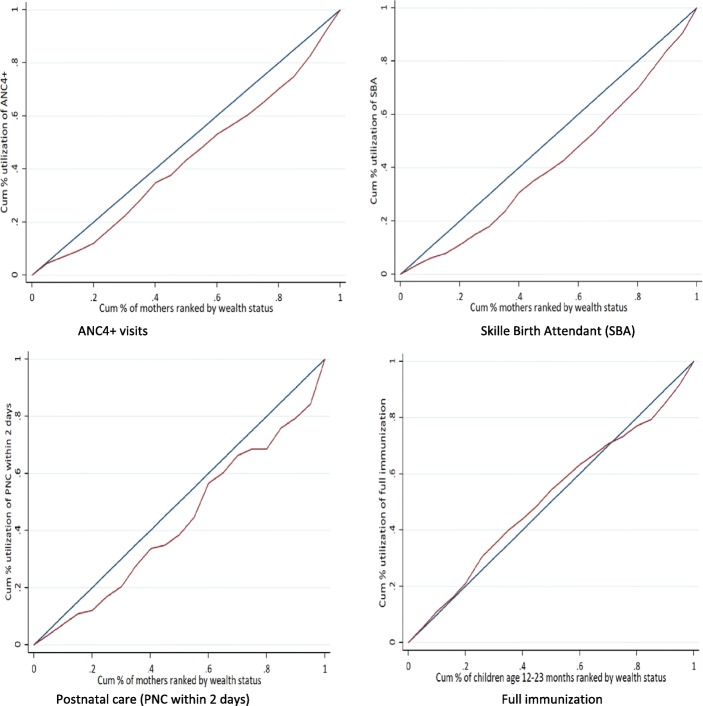
Concentration curve for selected maternal and child health services by wealth quintiles

The analysis of relative difference (Q5: Q1) showed that women from the better-off group were 2.2, 2.5 and 2.2 times more likely to have utilized ANC4+ visits, SBA and PNC services, respectively, compared to the poorest wealth quintile (Table 2). Correspondingly, the positive value of CIX for ANC4+, SBA, and PNC were 13.2, 17.5, and 14.1%, respectively, which indicated that there was inequity in the utilization of the services with the better-off mothers utilizing the services more than the poorest (Table 2 and Fig. 1).

### Child health services utilization

**Fig. 2:**
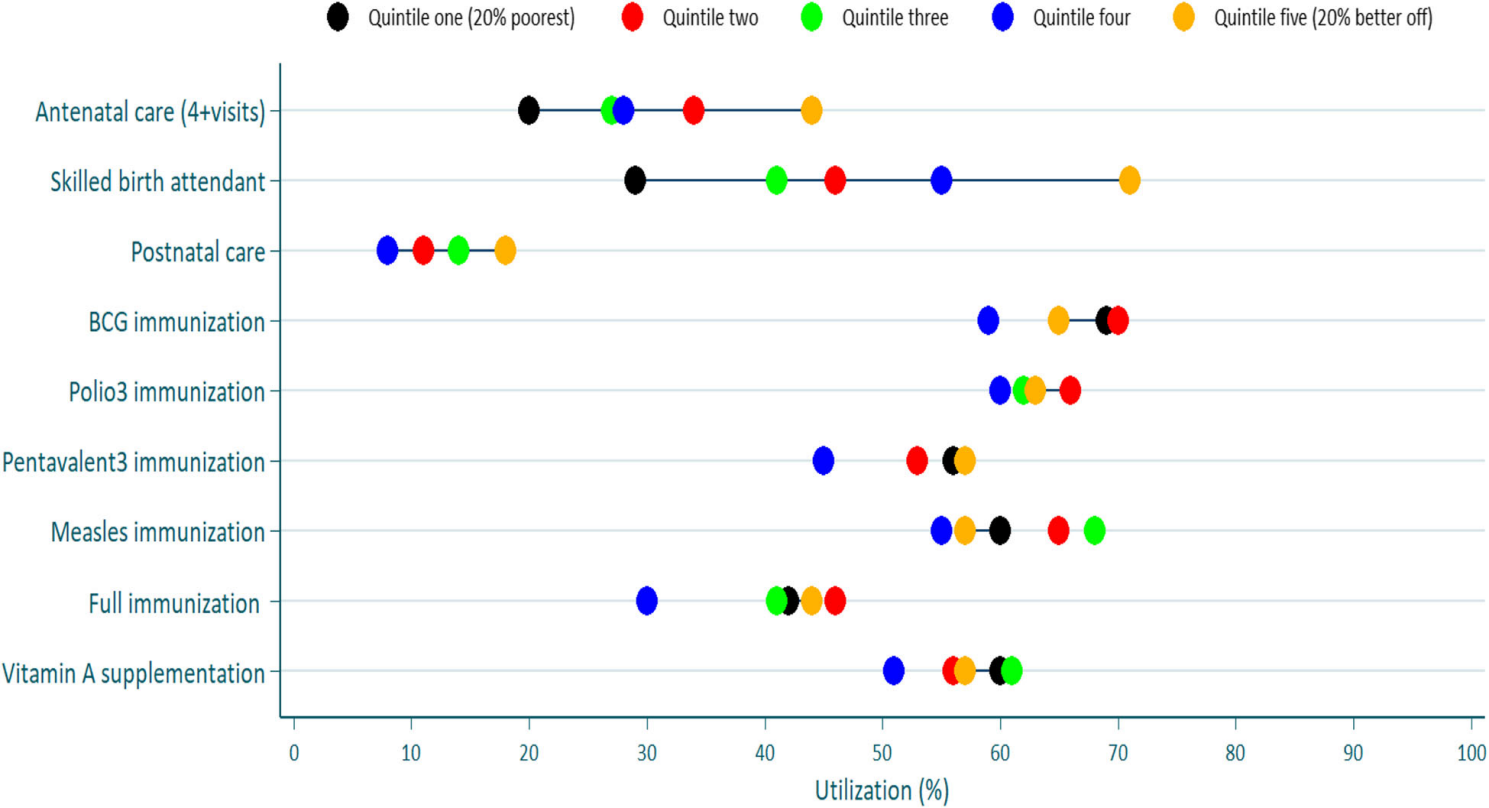
Percent utilization of maternal, neonatal, and child health services in each wealth quintiles Note: Colored dots show the mean coverage in each wealth quintile. Q1 represents the20% poorest wealth quintile and Q5 the 20% better-off. The distance between quintiles 1 and 5 represents absolute inequity. The horizontal lines connect the better-off (gold) and poorest (black) quintiles. The longer the line between the two groups, the greater the absolute inequity

Table 2 and Fig. 2 present the overall distribution in utilization of the six selected child health services and the inequity indices for these services. Close to two-thirds of children aged 12–23 months had received polio 3 and BCG immunizations, 54%of the children had received pentavalent 3, and 61% had measles immunization. Only 41% had been fully vaccinated.

The absolute inequity for all four basic immunizations showed slight differences in the utilization with a value of − 4 percentage points for BCG immunization, − 3 percentage points for polio 3, − 1 percentage points for pentavalent 3, − 3 percentage points for measles, and − 5 percentage points for full immunization. This implies that utilization of the four basic immunization types was in favor of the poorest wealth quintiles but with very small differences. Similarly, the slope index of inequity was negative for all the vaccination types, indicating that the utilization was slightly higher among individuals in the poorest wealth quintiles. But, the relative inequity (Q5: Q1) did not show any inequity in the utilization of these immunizations across the better-off and poorest children as the values for all are one or close to one (Table 2).

The concentration index for all the basic immunizations: BCG (− 2.4%), Polio 3 (− 1.8%), Pentavalent 3 (− 0.3%), and measles (− 2.1%) did not show any statistically significant differences in the utilization of all the four basic immunizations (Table 2). Also, the negative values of the slope index of inequity (− 5.0 percentage points) and concentration index (− 1.8%) in the utilization of full immunization were small (Table 2).

The absolute inequity results for vitamin A supplementation in children 6–23 months of age indicated that there was a small gap of − 3 percentage points, with a utilization rate of 60% in the poorest and 57% in the better-off. The concentration index (− 0.7%) and slope index of inequity (− 5.1 percentage points) indicated that the utilization of this service was concentrated among children in the poor wealth quintiles (Table 2).

Figure 2 illustrates the percent utilization and distribution of the maternal and child health services by wealth quintiles. ANC4+ visits, SBA and PNC (within two days) displayed a wide gap in utilization by wealth quintiles. This implies that maternal health services utilization was higher in the better-off. No immunizations showed differences in utilization by wealth quintiles. The distance between dots for all immunization types and vitamin A supplementation was close, indicating that the utilization of these services was more-or-less equitable.

## Discussion

This study showed mixed results regarding equity in the utilization of the selected maternal and child health services. The maternal health services utilization was inequitably distributed across the socioeconomic groups, with the better-off mothers being more likely to use these services. Having a skilled attendant at birth was the most inequitably distributed service, whilst four or more antenatal care visits and post-natal care within two days were moderately inequitable. In contrast, the utilization of all the basic childhood immunizations for children aged 12–23 months and vitamin A supplementation tended to be equitably distributed between the poorest and better-off.

The overall level of utilization of maternal health services remained low, which is consistent with the recent Ethiopian Demographic and Health Survey 2016 results [13]. Also, the coverage of immunizations and vitamin A supplementation was suboptimal, though, with a slightly higher levels than the national-level EDHS 2016 report [13].

In this study, maternal health services utilization tended to be inequitably distributed favoring mothers from the better-off socioeconomic group. Level of services utilization and equity in services utilization are interrelated, in that a low level of utilization is associated with high inequity in the use of services [12, 17, 25].

Inequities in maternal health services utilization were documented in the recent Ethiopian Demographic and Health Survey 2016 results [13] and found also in other recent studies in Ethiopia [16, 18, 26]. This finding is also consistent with the multi-country and multi-year analysis from sub-Saharan African countries [3], which has reported inequity in the utilization of antenatal care visits and facility-based delivery favoring mothers from the better-off socioeconomic status. Similar results have also been reported from other sub-Sharan African countries, such as Benin [27], Burkina Faso [28], and Nigeria [29], and other low- and middle-income countries, such as Philippines [30], Afghanistan [31], and Bangladesh [32]. In contrast, studies in Pakistan [33] and Myanmar [34] found equitable distribution in the utilization of four or more antenatal care visits.

This study was conducted in a rural part of the country, where a majority of the households have a low economic status and the health care infrastructure is weak with limited access to maternal health services [18], which is a constraint especially for poor people to use these services. Maternal health services utilization could be attributed to direct medical and non-medical costs for accessing the services, transport costs [18, 25, 35] and opportunity costs of the services [36] which cannot be afforded by the poorest women despite the fact that maternal health services are provided free to all mothers in Ethiopia [18, 27]. Women from higher socioeconomic groups may be empowered, have more autonomy and access to more economic resources than their poorer counterparts to use these services [36]. The health extension workers are responsible for linking mothers to health centers and hospitals but do not provide these maternal health services directly [37–39].

The utilization of all the studied childhood immunizations for children aged 12–23 months tended to be equitably distributed between the poorest and better-off. This is in line with findings from other studies in Ethiopia [4] and Pakistan [33]. In contrast, the Ethiopian Demographic and Health Survey 2016 [13], as well as reports from other low-and middle-income countries [21, 36], have shown immunization coverage in favor of the better-off. This difference in findings can partly be explained by the difference in samples.

This study was conducted in rural areas with a majority of the population being economically poor, having lower education levels, and where access to facility-based services was limited. The EDHS [13] was based on a nationally representative sample that included urban settings (e.g., Addis Ababa) with a high immunization coverage (89%) and rural and pastoralist areas (e.g., Afar) with least coverage (15%). This can explain the differences in findings of equity in full immunization between our results and the EDHS 2016.

In the rural parts of Ethiopia, the routine child immunization services are provided through outreach services by the health extension workers and occasionally via national immunization campaigns [26]. The outreach services for vaccination are carried out once a month, at religious holidays and sometimes through campaigns targeting the rural poor. The health extension workers were given this task based on the principles of primary health care [4] to provide community-based services (e.g., immunization services) to improve equitable access to all population groups [16]. This service provision is also supported by the women’s development groups, who are volunteer community health workers engaged in mobilizing mothers for immunization and other maternal and child health activities [38, 40]. Evidence shows that health extension workers are more effective in the provision of immunization services than maternal health services [39].

In this study, vitamin A supplementation tended to be equitably distributed among all children aged 12–23, which is expected as this supplementation is provided through campaigns to all children irrespective of their socioeconomic status. Though, this finding differs from the Ethiopian Demographic and Health Survey 2016 results.

In general, services delivered at fixed-level facilities, that are at health centers and hospitals, are more likely to be utilized by the better-off mothers [12, 31, 35] compared to the poorest mothers. This is the case for maternal health services in the Ethiopian healthcare delivery system [18], which is organized into three-tiered; the primary, secondary, and tertiary-level of care [10]. Services such as immunization and vitamin A supplementation that is close to the community and delivered through outreach services and campaigns by trained health extension workers, which are supported by women’s development groups are more likely to be equitably distributed [12, 35].

The inequity shown in the utilization of maternal health services implies that ensuring equitable and universal coverage of maternal health services is a challenge for Ethiopia. And attaining the maternal, neonatal, and child health targets in the SDGs would be unlikely. Moreover, to improve overall coverage of high-quality services, interventions that target the poorest [4] may be needed. This may include efforts to reduce financial barriers for poor mothers [25, 41].

This study has strengths and limitations. The findings from this study are relevant for the study areas in the four regions in the country that represent the selected intervention and comparison areas. Although not being sampled to represent the four regions, the level of utilization of maternal and child health services was similar to those found by the Ethiopian Demographic and Health Survey 2016 [13] and other recent studies [16, 18, 42]. The retrospective reporting of services utilization during pregnancy and newborns periods would possibly be affected by recall bias. The studied maternal services were, however, in their recent pregnancy i.e., by analyzing data restricted to births occurring in the 12 months prior to the survey. The results on child immunizations, which were based on interviews with caregivers, would also potentially suffer from reporting bias. The information relies on caregivers’ ability to accurately recall and report immunization, sometimes but not always supported by information in the immunization card. But the information on immunization used the same methodology as in the Demographic and Health Surveys. The equity assessment in this study included a range of selected maternal and child health services and was not limited to a single service indicator. We used a household wealth index to represent the social conditions of the families, while other characteristics, such as education level, urban or rural residency, could have revealed other nuances of equity in health service utilization.

## Conclusions

Utilization of four or more antenatal care visits, skilled birth attendance, and postnatal care within two days was low and inequitably distributed. In contrast, the utilization of immunizations and vitamin A supplementation was equitably distributed but with an overall low coverage. Although increasing the overall coverage of services may also increase equity in utilization, the pro-poor and pro-rural strategies of Ethiopia need to be reinforced in order to overcome the social gaps in the use of maternal, neonatal and child health services and eventually contribute to the achievement of SDGs. Monitoring the progress of SDGs over time and evaluating the health system investment using a combination of equity indices is critical for the country.

## Data Availability

The complete dataset will be available from the data repository center of EPHI upon request.

## Abbreviations

ANC4 +: Antenatal care visits 4 or more
BCG: Bacilli Calmette-Guérin
CBNC: Community Based Newborn Care
CIX: concentration index in its relative form
EDHS: Ethiopian Demography and Health Survey
HEP: Health extension program
HEW: Health extension workers
iCCM: Integrated Community Case Management of Childhood Illnesses
MICS: Multi-Indicator Cluster Survey
MNCH: Maternal, Neonatal, and Child Health
OHEP: Optimizing Health Extension Program
PCA: Principal Component Analysis
PNC: Postnatal Care
SBA: Skilled Birth Attendance
SDGs: Sustainable Development Goals
SII: Slope index of
SNNP: South Nations Nationalities Peoples Region
SSA: Sub-Saharan African
